# Patterns of Medication Prescription among Children and Adolescents with Attention-Deficit/Hyperactivity Disorder in the United States

**DOI:** 10.3390/children9020171

**Published:** 2022-01-30

**Authors:** Abdulkarim M. Meraya

**Affiliations:** 1 Department of Clinical Pharmacy, College of Pharmacy, Jazan University, Jazan 45124, Saudi Arabia; ameraya@jazanu.edu.sa; 2 Pharmacy Practice Research Unit, College of Pharmacy, Jazan University, Jazan 45124, Saudi Arabia

**Keywords:** attention-deficit/hyperactivity disorder, children, adolescents, prescription medications

## Abstract

The objectives of this study are to: (1) quantify the difference in the annual number of prescription medications (total and unique) between children and adolescents with ADHD and those without ADHD; and (2) identify the most prescribed medication classes and unique medications among children and adolescents with ADHD. A retrospective cross-sectional study design was employed using data from the 2015 and 2017 Medical Expenditure Panel Survey. The study sample comprised children and adolescents (5–17 years). In the 5–12-year age group, those with ADHD were 2.4%, 17%, and 15% significantly more likely to have one, 2–4, and ≥5 prescription medications, respectively. Similarly, those in the 13–17-year age group were more likely to have one prescription medication (3%), 2–4 prescription medications (15%), and ≥5 prescription medications (12%) than those without ADHD. The most prescribed medications among children and adolescents with ADHD were methylphenidate and amphetamine-dextroamphetamine. ADHD was associated with both higher annual total and unique prescription medications. Additionally, concurrent use of prescription medications was more prevalent among children and adolescents with ADHD. High-quality randomized clinical trials on the safety and efficacy of combinations of multiple psychotherapeutics and stimulants’ agents are required to guide the evidence-based practices.

## 1. Introduction

Attention-deficit/hyperactivity disorder (ADHD) is the most prevalent mental disorder among children and adolescents [1,2]. Approximately 9.4–10.2% of children and adolescents in the United States (US) have ADHD [1,2]. In addition, comorbid mental disorders are common among such children and adolescents [1,3]. In the US, 60% of children and adolescents with ADHD have at least one additional mental disorder, including behavior and conduct disorders, anxiety, depression, and autism spectrum disorders [1]. Children and adolescents with ADHD are also more likely to have other chronic physical conditions such as obesity [4], type 2 diabetes [5], and hypertension [6]. ADHD in children and adolescents is also associated with high healthcare expenditures [7] and a high number of medications [1,8].

In 2017, Gupte-Singh et al. used a sample of US children and adolescents to estimate the adjusted direct medical expenditures associated with ADHD [7]. They found that ADHD was associated with USD 949.24 annual total incremental direct medical expenditures after controlling for age, sex, race, health insurance coverage, and region of residence. The adjusted incremental prescription medication costs accounted for the largest proportion of the total expenditures [7]. Danielson et al. reported that 62% of their sample of children and adolescents with ADHD were taking medications [1]. Furthermore, children and adolescents with ADHD were more likely to have psychotropic polypharmacy (≥2 psychotropic medications) than their counterparts without ADHD [8]. Nevertheless, little is known about the overall number of medications among children and adolescents in general and specifically among those with ADHD.

Pediatric polypharmacy (concurrent use of multiple medications) is common in inpatient settings and is associated with drug–drug interactions and drug-related problems [9]. In outpatient settings, previous studies indicate that psychotropic polypharmacy is common among children and adolescents with mental disorders [8,10]. Nevertheless, little is known about polypharmacy in general among children and adolescents in outpatient settings [11]. Feinstein et al. found that 35% of children in the Colorado fee-for-service Medicaid population were exposed to some degree of polypharmacy [11]. They also found that children with complex chronic conditions are more likely to experience high-duration polypharmacy in outpatient settings [11]. Furthermore, they were more likely to have potential drug–drug interactions [11]. Children and adolescents with ADHD are more likely to have comorbid chronic mental and physical conditions [1,4,5,6]. As a result, it is expected that they are more likely to be exposed to a higher number of prescription medications in outpatient settings. Therefore, the present study has the following objectives: (1) quantify the difference in the number of prescription medications (total and unique) between children and adolescents with ADHD and those without ADHD; and (2) identify the most prescribed medication classes and unique medications among children and adolescents with ADHD.

## 2. Materials and Methods

### 2.1. Data Source

The cross-sectional study design was employed, using data from the 2015 and 2017 Medical Expenditure Panel Survey (MEPS). Household, prescription medications, and medical conditions files from the MEPS were used. The MEPS is an annual household survey of the US non-institutionalized civilian population. Information on demographics, socioeconomic characteristics, and health insurance coverage was retrieved from the MEPS household file. Medical conditions reported by the participants were extracted from the household file or the medical conditions file. In MEPS, chronic conditions are identified by asking the participants to enumerate the conditions in the MEPS priority list, those leading to hospital visits (inpatient, outpatient, and emergency), conditions that bothered the participants or caused disability during the reference period. This information is available in the household file of the MEPS. The medical conditions file of the MEPS provides information on the household-reported medical conditions linked to the International Classifications of Diseases, tenth edition, Clinical Modification (ICD-10) code. The MEPS prescription medications file provides information about the therapeutic classes of medicines in connection with the Multum Lexicon database. Based on the recommendations of the Agency for Healthcare Research and quality, we used alternate years, 2015 and 2017, to avoid the repetition of data of the same participants. MEPS data are described in detail at https://www.meps.ahrq.gov/mepsweb/ (accessed on 1 December 2021).

### 2.2. Study Sample

**Figure 1:**
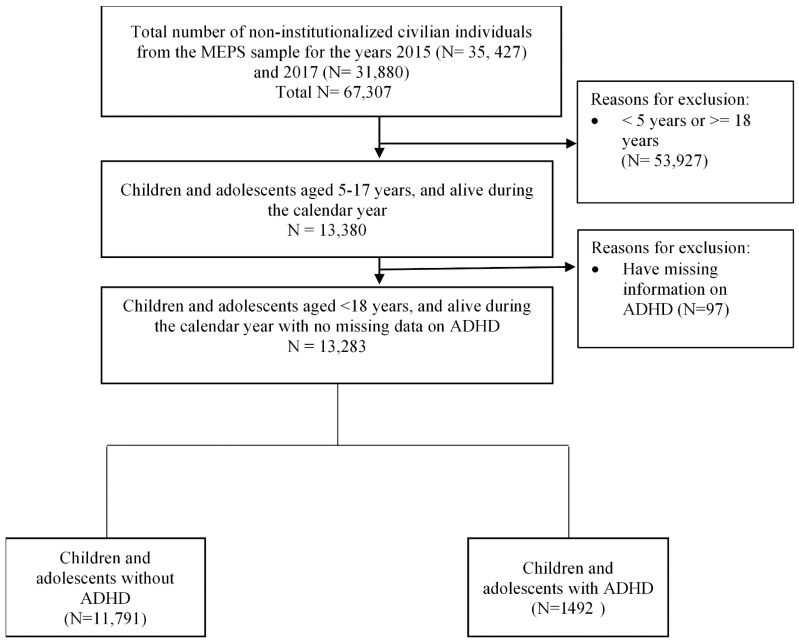
Flow diagram of study sample.

The study sample consisted solely of children and adolescents (5–17 years). In the 2015 and 2017 MEPS, there were 13,380 children aged between 5 and 17 years. Figure 1 shows the flowchart of the sample.

### 2.3. Measures

#### 2.3.1. Outcome

The annual total number of prescriptions was calculated for children and adolescents during the years of 2015 and 2017. Children and adolescents were classified into four mutually exclusive groups: (1) no prescription medication; (2) one prescription medication; (3) 2–4 prescription medications; (4) ≥5 prescription medications. Likewise, the annual total unique prescription medications were calculated for the children and adolescents in the study sample. Following a previous study [12], prescription medication use was classified into chronic use (≥30 days) and acute use (<30 days) based on the days’ supply of medication. All calculations were computed using the unique generic names in the MEPS prescription medication file.

#### 2.3.2. Key Explanatory Variable

In this study, children and adolescents were classified into two groups: (1) ADHD: Children and adolescents who were reported as having the disease in the household file or those with the F90 International Classifications of Diseases, tenth edition, Clinical Modification (ICD-10) code in the medical conditions file; and (2) no ADHD.

#### 2.3.3. Other Explanatory Variables

The other explanatory variables included sex (female, male); age (5–12 and 13–17 years); race/ethnicity (White, African American, Latino, other); poverty status: poor (<100% federal poverty line), near poor (100% to <200%), middle income (200% to <400%), and high income (≥400%); health insurance coverage (private, public, uninsured); number of other co-occurring physical conditions (adverse shock/allergic reactions, asthma, bronchitis, cancer, cardiac dysrhythmias, disorders of lipid metabolism, dermatitis/eczema, diabetes, epilepsy, gastroesophageal reflux disease, hypertension, joint disorders, migraine, obesity, pain unspecified, thyroid disorders); and number of other co-occurring mental conditions (anxiety, bipolar disorder, major depression, mood disorders, pervasive developmental disorders, stress). Appendix A lists the diagnosis codes of the physical and mental conditions included in this study.

### 2.4. Statistical Techniques

Chi-square tests were used to examine the associations between the studied groups and other explanatory variables in the bivariate analysis. Due to the ordinal nature of the outcome, an ordered probit regression model was computed to estimate the probability of being in any prescription medication group. The key assumption in the ordered probit regression model is the assumption of parallel lines, which assumes that the effects of any explanatory variables are consistent across the different cut-offs or thresholds. The Wald test of parallel lines assumption for the final model was used by employing the gologit2 function in STATA 15.1, and the results indicated that the final model does not violate the proportional odds/parallel lines assumption [13]. The marginal effects of all explanatory variables on the ordered number of prescription medication probabilities were computed. All analyses were stratified by age (5–12 and 13–17 years). In all models, the reference group was “children and adolescents with no ADHD”. In the adjusted models, the following variables were controlled for sex, race/ethnicity, poverty status, health insurance coverage, and number of co-occurring physical and mental health conditions. All analyses were conducted using survey procedures in STATA 15.1.

## 3. Results

### 3.1. Description of the Study Sample

The study sample consisted of 13,283 children and adolescents aged 5–17 years. Of the sample, 60% were aged 5–12 years. Approximately 51% of the children and adolescents were male and 12% had ADHD. Around half of the children and adolescents were White (49.6%), and most had private insurance (60%). Most of the study sample had no chronic physical conditions (82.1%) or chronic mental conditions (92.8%).

In the 5–12-year age group, a significantly higher percentage of those with ADHD were male (69% vs. 49.4%), were White (56.5% vs. 48.5%), were poor (26.7% vs. 18.9%), had public insurance (51.3% vs. 38.1%), had one physical condition (19.0% vs. 13.6%), and had one mental condition (18.0% vs. 3.3%) as compared to those without ADHD.

In the 13–17-year age group, a significantly higher proportion of those with ADHD were male (69.5% vs. 46.8%), were White (58.7% vs. 48.4%), were poor (19.3% vs. 14.1%), had public insurance (41.9% vs. 31.8%), and had one mental condition (12.7% vs. 5.6%) as compared to their counterparts without ADHD. Table 1 shows the characteristics of the studied children and adolescents by age group and ADHD status.

### 3.2. ADHD and Prescription Medications

Table 1 shows the proportions of the prescription medication number among the children and adolescents by age group and ADHD status. Overall, 58.1% of the children and adolescents in the sample did not have any prescription medication during the reference period. Approximately 21% of them had one unique prescription medication, 18% had 2–4 unique prescription medications, and 3.2% had ≥5 unique prescription medications. Of the sample, 33% were issued at least one prescription medication for ≥30 days.

In the 5–12-year age group, a significantly higher proportion of those with ADHD had ≥2 concurrent prescription medications within a round than those without ADHD. Similarly, a higher proportion of those in the 13–17-year age group with ADHD had 2–4 concurrent prescription medications (33.9% vs. 15.3%) and ≥5 concurrent prescription medications (4.9% vs. 1.0%).

Table 2 shows the weighted prevalence in the annual use of prescription medications by therapeutic medication class among the children and adolescents overall and by ADHD status. The most prescribed therapeutic medication classes were anti-infective (26.2%). Specifically, penicillins were the most prescribed medications (13.6%). The second most prescribed therapeutic medication class was central nervous system (CNS) agents (18.7%), followed by respiratory agents (16.7%).

A higher proportion of the children and adolescents with ADHD were prescribed CNS agents (37.8% vs. 12.3%), psychotherapeutic agents (11.2% vs. 2.7%), and cardiovascular agents (8.0% vs. 1.8%) than those without ADHD. Furthermore, 6.3% of children and adolescents with ADHD were prescribed antiadrenergic agents; 29.2% were prescribed CNS stimulants; and 7.2% were prescribed antidepressants.

In the entire sample, the most prescribed medication was amoxicillin (N = 1110), followed by albuterol (N = 792), ibuprofen (N = 370), methylphenidate (N = 312), and montelukast (N = 272). Among the children and adolescents with ADHD, the most prescribed medication was methylphenidate (N = 304), followed by amphetamine-dextroamphetamine (N = 176), lisdexamfetamine (N = 142), amoxicillin (N = 112), albuterol (N = 128), and guanfacine (N = 112).

### 3.3. ADHD and Number of Prescription Medications

Table 3 summarizes the estimated marginal effect of ADHD status and the other explanatory variables on the probabilities of the annual prescription medication number from the ordered probit models. Those in the 5–12-year age group with ADHD were 34% significantly less likely to have no prescription medication. Nevertheless, they were 2.4%, 17%, and 15% more likely to have one, 2–4, and ≥5 prescription medications, respectively. Similarly, those in the 13–17-year age group with ADHD were less likely to have no prescription medication (30%). However, they were more likely to have one prescription medication (3%), 2–4 prescription medications (15%), and ≥5 prescription medications (12%) than those without ADHD.

### 3.4. Other Explanatory Variables and Number of Prescription Medications

There was a significant relationship between sex and prescription medication number only for the 13–17-year age group. Males were more likely to have more prescription medications than females. In the 13–17-year age group, African Americans, Hispanics, and other races were less likely than White children and adolescents to have prescription medications.

There was no relationship between poverty status and prescription medication use among children and adolescents in the study sample. In the 5–17-year age group, those with public insurance were more likely to have one prescription medication (1%), 2–4 prescription medications (2.4%), and ≥5 prescription medications (1.4%) than those with private insurance. Conversely, uninsured children and adolescents were less likely to have a high number of prescription medications than those with private insurance.

Overall, children and adolescents with ≥1 chronic physical condition were more likely to have a high number of prescription medications. Specifically, those aged 5–12 years were more likely to have one prescription medication (3.8%), 2–4 prescription medications (19.8%), and ≥5 prescription medications (13.7%) than those with no chronic physical conditions. Moreover, children and adolescents with ≥2 chronic physical conditions were more likely to have 2–4 prescription medications (23.9%) and ≥5 prescription medications (31%) than those with no chronic physical conditions. Additionally, older children and adolescents with ≥1 chronic physical condition were more likely to have a high number of prescription medications. For example, children and adolescents with two chronic physical conditions were more likely to have one prescription medication (2.4%), 2–4 prescription medications (22.8%), and ≥5 prescription medications (36.4%) than those with no chronic physical conditions. However, those aged 13–17 years were less likely to have no prescription medication (57%) than those with no chronic physical conditions.

Similarly, children and adolescents with chronic mental conditions were more likely to have a high number of prescription medications across the age groups. For example, those aged 5–12 years with ≥2 chronic mental conditions were more likely to have 2–4 prescription medications (16.3%) and ≥5 prescription medications (17.4%) than those with no chronic mental conditions. Furthermore, those aged 13–17 years with two chronic mental conditions were more likely to have 2–4 prescription medications (18.4%) and ≥5 prescription medications (24.5%).

## 4. Discussion

The present study evaluated the difference in the number of prescription medications (both total and unique) between children and adolescents with ADHD and those without ADHD. In this study, children and adolescents with ADHD were more likely to have a higher number of prescription medications. The most prescribed medication class among the children and adolescents with ADHD was CNS stimulants. Furthermore, 7.5% of all children and adolescents in this study used CNS stimulants. Zuvekas et al. [14] studied a representative sample of US children and adolescents, and found that 4.8% of those aged 6–12 years old were prescribed CNS stimulants in 2002. The results of the present study and that by Zuvekas et al. suggest the increased utilization of CNS stimulants among children and adolescents in the US. CNS stimulants are effective and safe treatments for ADHD [15]. However, they are also associated with various adverse events [16] including rare cardiovascular events [17]. Therefore, more studies are required for examining the short-term and long-term adverse effects of CNS stimulants among children and adolescents with ADHD. In the present study, the overall prevalence of ADHD was 12.1%. This prevalence was higher than the prevalence estimated (7.8%) by Visser et al., who used a national sample of children and adolescents in the US in 2003 [18].

In the present study, chronic mental conditions were more prevalent among children and adolescents with ADHD. Furthermore, children and adolescents with ADHD were more likely to be given psychotherapeutic medications. They also had a higher number of unique concurrent prescription medications within a round than children and adolescents without ADHD. ADHD is a multifactorial and complex disease, and is associated with multiple symptoms, including inattention, hyperactivity, and impulsivity [19]. There are some benefits of using multiple psychotropic medications for treating ADHD [20,21]. Therefore, numerous studies have found that the use of multiple psychotropic medications is common among children and adolescents in the US and Europe [21,22,23,24]. Nevertheless, more than half of the children and adolescents with ADHD have at least one additional chronic mental condition [1]. This also could explain the high use of psychotherapeutic agents, especially antidepressants, among children and adolescents with ADHD. It should be noted that the safety of these combinations is unclear [20,21]. Therefore, high-quality randomized controlled trials are needed to assess the efficacy and safety of using multiple psychotropic medications among children and adolescents with ADHD.

Of all children and adolescents in the present study, 18.6% had ≥2 chronic prescription medications. Moreover, 18.4% of them were prescribed ≥2 unique concurrent prescription medications within a round. Children and adolescents are a vulnerable population and are often excluded from clinical trials. Therefore, the safety of medications and their long-term effects on children and adolescents are often unclear. Concurrent use of medications in children and adolescents puts them at higher risk for adverse events and drug–drug interactions. Qato et al. estimated that 8.2% of their sample of children and adolescents who used ≥2 concurrent prescription medications were at risk for potential major drug–drug interactions [12]. They also found that half of the interacting medications included psychotropic medications (primarily antidepressants) [12]. Physicians and other independent prescribers should closely monitor prescription medications for children and adolescents, especially for those with chronic physical or mental conditions. Furthermore, communications between independent prescribers and an integration of health care can play an important role in minimizing the risk for adverse events and potential drug–drug interactions among children and adolescents. Pharmacists also can play an important role in minimizing unwanted medications’ outcomes by reviewing medications’ history to inform prescribers of potential drug–drug interactions.

In this study, uninsured children and adolescents were more likely to have no prescription medications, and they were less likely to have a high number of prescription medications. Furthermore, children with public insurance were more likely to have a high number of prescription medications. Previous studies indicated that children insured by Medicaid and the Children’s Health Insurance Program were significantly more likely to receive preventive medical and dental routine care as compared to their counterparts with private insurance [25,26]. Policy makers need to address the access to care among children with private insurance to improve health outcomes in this population.

The results should be interpreted in the context of some limitations. The MEPS does not provide information on disease severity, which may affect the total number of prescription medications. Moreover, medication data were limited to prescription medications and did not include over-the-counter medications. Consequently, the rate of medications’ use may have been underestimated. Furthermore, all information in this study was self-reported and may be subject to recall bias and social desirability. Finally, this is a cross sectional study. Therefore, it is difficult to assess the temporal relationships between the explanatory variables and the outcomes.

## 5. Conclusions

ADHD was associated with both higher annual total and unique prescription medications. Additionally, concurrent use of prescription medications was more prevalent among children and adolescents with ADHD. High-quality randomized clinical trials on the safety and efficacy of combinations of multiple psychotherapeutics and stimulants’ agents are required to guide the evidence-based practices.

## Figures and Tables

**Table 1:** Characteristics of the study sample by age and the presence of Attention-deficit/hyperactivity disorder (N = 13,283).

Weighted Percentages Medical Expenditure Panel Survey, Panels 2015 and 2017							
		Age: 5–12 Years		*p*-Value	Age: 13–17 Years		*p*-Value
	Total	No ADHD	ADHD		No ADHD	ADHD	
	Wt. (%)	Wt. (%)	Wt. (%)		Wt. (%)	Wt. (%)	
All		89.3	10.7		85.8	14.2	
Sex				<0.001			<0.001
Female	49.1	50.6	31.0		53.2	30.5	
Male	50.9	49.4	69.0		46.8	69.5	
Race				<0.001			<0.001
White	49.6	48.5	56.5		48.4	58.7	
African American	14.0	13.7	17.1		13.5	15.5	
Latino	25.0	25.9	16.5		26.5	16.7	
Other	11.5	11.9	9.8		11.5	9.1	
Poverty status				<0.001			0.032
Poor	17.8	18.9	26.7		14.1	19.3	
Near Poor	21.4	21.4	23.8		20.9	22.2	
Middle Income	30.3	29.8	27.5		31.8	30.1	
High Income	30.4	29.8	22.0		33.2	28.4	
Health insurance				<0.001			<0.001
Private	60.0	59.3	47.2		64.0	56.4	
Public	37.0	38.1	51.3		31.8	41.9	
Uninsured	3.0	2.6	1.5		4.2	1.7	
Chronic Physical Conditions Number				<0.001			0.076
No Physical condition	82.1	82.9	74.5		82.8	78.7	
One physical condition	14.1	13.6	19.0		13.4	17.5	
≥2	3.8	3.5	6.5		3.8	3.7	
Chronic Mental Conditions Number				<0.001			<0.001
No mental condition	92.8	96.5	77.7		92.7	75.9	
One mental condition	5.6	3.3	18.0		5.6	12.7	
≥2	1.6	0.2	4.3		1.6	11.4	
Region of residence				0.004			0.033
Northeast	16.3	15.4	20.1		16.9	15.9	
Midwest	21.5	21.4	24.8		20.9	21.7	
South	38.2	39.3	38.4		35.8	42.9	
West	24.0	23.9	16.8		26.4	19.5	
Total prescription medication use				<0.001			<0.001
No medications	58.1	61.8	24.1		63.6	28.1	
1 medication	16.3	18.1	10.3		14.4	17.2	
2–4 medications	18.5	15.6	41.0		16.8	30.9	
≥5 medications	7.1	4.5	24.6		5.2	23.7	
Total unique prescription medication use				<0.001			<0.001
No medications	58.1	61.8	24.1		63.6	28.1	
1 medication	20.7	21.0	26.0		17.7	29.6	
2–4 medications	18.1	14.9	40.3		16.1	34.2	
≥5 medications	3.2	2.2	9.6		2.6	8.1	
Total chronic prescription medication use				<0.001			<0.001
No medications	67.0	70.0	27.9		75.0	35.0	
1 medication	14.4	16.2	11.0		11.7	16.4	
2–4 medications	14.6	11.6	42.7		11.2	31.3	
≥5 medications	4.0	2.1	18.4		2.1	17.3	
Highest Number of prescription medications within a round				<0.001			<0.001
No medications	58.1	61.8	24.1		63.6	28.1	
1 medication	23.6	23.8	31.7		20.2	33.1	
2–4 medications	16.8	13.4	38.3		15.3	33.9	
≥5 medications	1.6	1.1	5.8		1.0	4.9	

ADHD: Attention-deficit/hyperactivity disorder; Wt.: weighted. Note: Based on 13,380 children and adolescents aged 5–17 years. The *p*-values were derived from chi-square tests between children and adolescents with ADHD vs. those without. Chronic physical conditions include adverse shock/allergic reactions, asthma, bronchitis, cancer, cardiac dysrhythmias, disorders of lipid metabolism, dermatitis/eczema, diabetes, epilepsy, gastroesophageal reflux disease, hypertension, joint disorders, migraine, obesity, pain unspecified, and thyroid disorders. Chronic mental conditions include anxiety, bipolar disorder, major depression, mood disorders, pervasive developmental disorders, and stress.

**Table 2:** Weighted prevalence in the annual use of prescription medications by therapeutic drug class among children and adolescents, overall and by ADHD status.

Medical Expenditure Panel Survey, Panels 2015 and 2017				
		Overall, N = 5168	No ADHD, N = 4110	ADHD, N = 1058
Anti-infectives		26.2 (24.95–27.44)	30.4 (29–31.8)	13.5 (11.5–15.5)
	Amebicides	1.05 (0.78–1.32)	1.25 (0.79–1.7)	0.51 (0.07–0.95)
	Antifungals	0.26 (0.14–0.38)	1.23 (0.9–1.57)	0.32 (0.02–0.61)
	Antimalarial	0.6 (0.36–0.84)	0.24 (0.11–0.36)	0.45 (0.09–0.8)
	Antiviral	2.25 (1.79–2.7)	0.65 (0.37–0.94)	1.35 (0.59–2.11)
	Cephalosporins	2.72 (2.21–3.24)	2.55 (1.99–3.1)	1.35 (0.45–2.25)
	Macrolide	3.24 (2.76–3.73)	3.19 (2.58–3.8)	2.1 (1.31–2.89)
	Miscellaneous Antibiotics	1.02 (0.75–1.28)	3.63 (3.07–4.18)	0.58 (0.26–0.9)
	Penicillins	13.61 (12.67–14.55)	1.16 (0.83–1.5)	5.95 (4.78–7.11)
	Quinolones	0.32 (0.09–0.55)	16.18 (15.05–17.32)	0.20 (0–0.4)
	Sulfonamides	0.35 (0.16–0.54)	0.32 (0.17–0.47)	0.44 (0–1.06)
	Tetracyclines	0.22 (0.12–0.32)	0.32 (0.17–0.47)	0.09 (0–0.26)
	Urinary Anti-infectives	0.09 (0.02–0.17)	0.27 (0.13–0.4)	0.09 (0–0.23)
	Lincomycin Derivatives	1.05 (0.78–1.32)	0.47 (0.24–0.71)	0.23 (0.02–0.44)
Antineoplastic		0.65 (0.41–0.89)	0.80 (0.5–1.1)	0.30 (0–0.5)
	Antimetabolites	0.06 (0.01–0.12)	0.07 (0–0.14)	0.04 (0–0.13)
	Antineoplastic Hormones	0.15 (0.04–0.26)	0.18 (0.04–0.31)	0.08 (0–0.19)
	Miscellaneous Antineoplastic	0.34 (0.18–0.51)	0.43 (0.21–0.64)	0.10 (0–0.28)
Cardiovascular Agents		3.31 (2.8–3.81)	1.8 (1.3–2.2)	8.0 (6.5–9.4)
	Angiotensin Converting Enzyme Inhibitors	0.03 (0–0.05)	0 (0–0)	0.04 (0–0.13)
	Antiadrenergic Acting Peripherally	0.02 (0–0.04)	0.04 (−0.01–0.1)	0.03 (0–0.1)
	Antiadrenergic Acting Centrally	1.71 (1.38–2.05)	0.09 (0–0.17)	6.32 (5.1–7.53)
	Antiarrhythmic	0.04 (0–0.08)	0.08 (0–0.17)	0.03 (0–0.08)
	Beta Adrenergic Blocking	0.09 (0.01–0.16)	0.09 (0–0.17)	0.10 (0–0.24)
	Calcium Channel Blocking	0.09 (0.01–0.17)	0 (0–0)	0.06 (0–0.17)
	Vasopressors	0.82 (0.57–1.07)	0.96 (0.65–1.27)	0.42 (0–1.02)
	Angiotensin II inhibitors	0.02 (0–0.05)	0.01 (0–0.02)	0.05 (0–0.16)
	Anticholinergic Chronotropic	0.01 (−0.01–0.03)	0 (0–0)	0.03 (0–0.1)
Central Nervous System agents		18.68 (17.64–19.73)	12.3 (11.3–13.4)	37.8 (35.1–40.5)
	Analgesics	6.21 (5.52–6.91)	7.29 (6.47–8.12)	3.01 (2.16–3.85)
	Anticonvulsants	1.22 (0.92–1.51)	0.88 (0.61–1.15)	2.21 (1.5–2.92)
	Antiemetic/Antivertigo Agents	2.1 (1.79–2.41)	2.39 (1.99–2.8)	1.21 (0.73–1.69)
	Antiparkinson Agents	0.02 (−0.01–0.05)	0.01 (−0.01–0.04)	0.05 (0–0.14)
	Anxiolytics, Sedatives, and Hypnotics	0.88 (0.65–1.12)	0.69 (0.45–0.93)	1.46 (0.88–2.03)
	CNS Stimulants	7.59 (6.8–8.38)	0.31 (0.09–0.53)	29.23 (26.76–31.7)
	Muscle Relaxants	0.33 (0.12–0.55)	0.29 (0.06–0.51)	0.47 (0–1.01)
Gastrointestinal Agents		2.6 (2.2–3)	2.9 (2.5–3.4)	1.6 (0.9–2.3)
	Antacids	0.08 (0.03–0.13)	0.08 (0.03–0.14)	0.07 (−0.02–0.16)
	Antidiarrheals	0.09 (0.04–0.15)	0.12 (0.04–0.19)	0.02 (−0.02–0.07)
	H2 Antagonists	0.53 (0.37–0.69)	0.56 (0.37–0.74)	0.45 (0.12–0.77)
	Laxatives	0.8 (0.58–1.02)	0.94 (0.67–1.21)	0.4 (0.1–0.7)
	Proton Pump Inhibitors	0.77 (0.56–0.98)	0.85 (0.61–1.09)	0.54 (0.13–0.96)
	Functional Bowel Disorder Agents	0.16 (0.09–0.24)	0.2 (0.11–0.3)	0.04 (0–0.13)
Hormones/Hormone Modifiers		5.63 (5.01–6.25)	6.3 (5.6–7.1)	3.6 (2.6–4.5)
	Adrenal Cortical Steroids	3.09 (2.68–3.51)	3.42 (2.94–3.89)	2.13 (1.36–2.9)
	Sex Hormones	1.35 (1.04–1.65)	1.62 (1.23–2.01)	0.53 (0.19–0.86)
	Thyroid Hormones	0.31 (0.17–0.45)	0.33 (0.17–0.49)	0.25 (0.01–0.49)
Miscellaneous Agents		0.56 (0.29–0.82)	0.40 (0.20–0.70)	1 (0.4–1.6)
	Smoking Cessation	0.47 (0.24–0.70)	0.34 (0.11–0.56)	0.85 (0.32–1.39)
Nutritional Products		2.60 (2.09–3.10)	2.9 (2.30–3.50)	1.7 (0.9–2.5)
	Iron Products	0.46 (0.26–0.66)	0.37 (0.22–0.53)	0.71 (0.06–1.35)
	Minerals and Electrolytes	1.04 (0.69–1.39)	1.26 (0.8–1.71)	0.39 (0.02–0.76)
	Vitamins	0.67 (0.46–0.89)	0.74 (0.47–1.01)	0.46 (0.16–0.77)
	Vitamin and Mineral Combinations	0.26 (0.14–0.38)	0.31 (0.16–0.46)	0.10 (0–0.24)
Respiratory Agents		16.74 (15.77–17.72)	18.4 (17.3–19.6)	11.7 (10.1–13.2)
	Antihistamines	4.7 (4.14–5.25)	5.08 (4.43–5.74)	3.54 (2.63–4.45)
	Antitussives	0.17 (0.08–0.26)	0.15 (0.06–0.25)	0.23 (0–0.45)
	Bronchodilators	7.59 (6.9–8.28)	8.4 (7.59–9.21)	5.2 (4.07–6.33)
	Decongestants	0.12 (0.01–0.23)	0.16 (0.01–0.31)	0.02 (0–0.05)
	Expectorants	0.37 (0.21–0.53)	0.45 (0.26–0.65)	0.13 (0.02–0.24)
	Respiratory Inhalant Products	1.75 (1.38–2.11)	1.83 (1.43–2.23)	1.5 (0.66–2.34)
	Upper Respiratory Combinations	1.85 (1.45–2.25)	2.12 (1.66–2.59)	1.04 (0.51–1.56)
	Leukotriene Modifiers	2.68 (2.25–3.11)	2.95 (2.4–3.49)	0.23 (0.02–0.44)
Topical Agents		15.51 (14.59–16.44)	18.1 (16.9–19.2)	7.8 (6.6–9)
	Antiseptic and Germicides	0.15 (0.08–0.23)	0.2 (0.09–0.3)	0.03 (0–0.08)
	Dermatological Agents	7.82 (7.18–8.46)	9.32 (8.5–10.13)	3.35 (2.55–4.15)
	Mouth and Throat Products	0.25 (0.11–0.39)	0.32 (0.14–0.51)	0.04 (0–0.13)
	Ophthalmic Preparations	2.04 (1.69–2.39)	2.34 (1.93–2.75)	1.17 (0.4–1.93)
	Otic preparations	1.3 (0.94–1.66)	1.56 (1.13–2)	0.53 (0.17–0.89)
	Nasal Preparations	2.91 (2.4–3.41)	3.25 (2.64–3.85)	1.89 (1.16–2.63)
Psychotherapeutic Agents		4.84 (4.17–5.52)	2.7 (2.1–3.4)	11.2 (9.5–12.8)
	Antidepressants	3.4 (2.85–3.95)	2.14 (1.58–2.7)	7.15 (5.76–8.54)
	Antipsychotics	0.03 (0–0.07)	0 (0–0)	0.1 (0–0.26)
Metabolic Agents		0.18 (0.06–0.29)	0.9 (0.4–1.3)	0.4 (0.1–0.7)
	Antihyperlipidemic	0.07 (0.02–0.13)	0.08 (0.01–0.14)	0.07 (0–0.17)
	Antidiabetics	0.09 (0.04–0.15)	0.7 (0.3–1.1)	0.23 (0.02–0.45)

ADHD: Attention-deficit/hyperactivity disorder.

**Table 3:** Estimated marginal effect of the explanatory variables on number of prescription medications probabilities.

		Age: 5–12 Years				Age: 13–17 Years			
		No Prescriptions	1 Prescription	2–4 Prescriptions	≥5 Prescriptions	No Prescriptions	1 Prescription	2–4 Prescriptions	≥5 Prescriptions
Explanatory Variables									
ADHD	No	Reference							
	Yes	−0.338 ***	0.024 ***	0.167 ***	0.147 ***	−0.296 ***	0.031 ***	0.146 ***	0.120 ***
Sex	Female	Reference							
	Male	−0.003	0.001	0.001	0.001	0.035 *	−0.008 *	−0.017 *	−0.010 *
Race	White	Reference							
	African Americans	0.097 ***	−0.024 ***	−0.047 ***	−0.026 ***	0.107 ***	−0.025 ***	−0.052 ***	−0.029 ***
	Hispanics	0.035	−0.008	−0.017	−0.010	0.058 **	−0.013 **	−0.029 **	−0.017 **
	Other	0.086 ***	−0.021 ***	−0.042 ***	−0.024 ***	0.083 **	−0.019 **	−0.041 **	−0.024 **
Poverty Status	Poor	Reference							
	Near Poor	−0.006	0.002	0.003	0.002	0.009	−0.002	−0.004	−0.002
	Middle Income	−0.017	0.004	0.008	0.005	−0.017	0.004	0.008	0.005
	High Income	−0.03	0.007	0.015	0.009	−0.045	0.01	0.022	0.013
Health insurance	Private	Reference							
	Public	−0.049 **	0.011 **	0.024 **	0.014 **	−0.025	0.005	0.012	0.007
	Uninsured	0.113 **	−0.033 **	−0.054 **	−0.026 **	0.102 *	−0.027 *	−0.05 *	−0.025 **
Chronic Physical Conditions Number	No physical condition	Reference							
	One physical condition	−0.372 ***	0.038 ***	0.198 ***	0.137 ***	−0.347 ***	0.042 ***	0.183 ***	0.122 ***
	≥2	−0.530 ***	−0.020	0.239 ***	0.311 ***	−0.569 ***	−0.024 *	0.228 ***	0.364 ***
Chronic Mental Conditions Number	No mental condition	Reference							
	One mental condition	−0.182 ***	0.027 ***	0.091 ***	0.065 ***	−0.250 ***	0.029 ***	0.123 ***	0.098 ***
	≥2	−0.351 ***	0.013	0.163 ***	0.174 ***	−0.430 ***	0.001	0.184 ***	0.245 ***
Region of Residence	Northeast	Reference							
	Midwest	−0.038	0.008	0.019	0.011	−0.014	0.003	0.007	0.004
	South	−0.033	0.007	0.016	0.010	−0.033	0.007	0.016	0.010
	West	0.050 *	−0.013 *	−0.024 *	−0.013	0.02	−0.005	−0.01	−0.005

ADHD: Attention-deficit/hyperactivity disorder. Note: Based on 13,380 children and adolescents aged 5–17 years. Chronic physical conditions include adverse shock/allergic reactions, asthma, bronchitis, cancer, cardiac dysrhythmias, disorders of lipid metabolism, dermatitis/eczema, diabetes, epilepsy, gastroesophageal reflux disease, hypertension, joint disorders, migraine, obesity, pain unspecified, and thyroid disorders. Chronic mental conditions include anxiety, bipolar disorder, major depression, mood disorders, pervasive developmental disorders, and stress. Asterisks represent significant group differences compared to the reference group based on the ordered probit regression model and the outcome number of prescription medications (No prescriptions, 1 prescription, 2–4 prescriptions, ≥5). *** *p* < 0.001; ** 0.001 ≤ *p* < 0.01; * 0.01 ≤ *p* < 0.05.

## Data Availability

This study was based on a publicly available dataset, MEPS, and can be obtained directly from www.meps.ahrq.gov (accessed on 1 December 2021).

## Appendix A. List of Chronic Physical and Mental Conditions Diagnosis Codes

**Disease**	**Diagnosis Codes 2015**	**Diagnosis Codes 2017**
Chronic Physical conditions		
Adverse Shock/Allergic Reactions	ICD-9-CM Code: 995.	
Asthma	Clinical Classification Code: 253.	ICD-10-CM Code: T78.
Bronchitis	Clinical Classification Code: 128.	ICD-10-CM Code: J45
Cancer	ICD-9-CM Code: 25, 466, 490, 491.	ICD-10-CM Code: J20, J40, J41, J42.
Cardiac dysrhythmias	Clinical Classification Code: 011, 012, 013, 014, 015, 016, 017, 018, 019, 020, 021, 022, 023, 024, 025, 026, 027, 028, 029, 030, 031, 033, 034, 035, 036, 037, 039, 040, 041, 042, 043, 044.	ICD-10-CM Code: C00-C97, D00-D09, D37-D48.
Disorders of lipid metabolism	Clinical Classification Code: 106.	ICD-10-CM Code: I49.
Dermatitis/Eczema	Clinical Classification Code: 053.	ICD-10-CM Code: E78.
Diabetes	ICD-9-CM Code: 691, 692.	ICD-10-CM Code: L20-L30.
Epilepsy	Clinical Classification Code: 049, 050.	ICD-10-CM Code: E11.
Gastroesophageal reflux disease	Clinical Classification Code: 083.	ICD-10-CM Code: G40.
Hypertension	Clinical Classification Code: 138.	ICD-10-CM Code: K21.
Joint Disorders	Clinical Classification Code: 98.	ICD-10-CM Code: I10.
Migraine	ICD-9-CM Code: 719.	ICD-10-CM Code: M24, M25.
Obesity	ICD-9-CM Code: 346.	ICD-10-CM Code: G43.
Pain Unspecified	ICD-9-CM Code: 278.	ICD-10-CM Code: E66.
Thyroid Disorders	ICD-9-CM Code: 338.	ICD-10-CM Code: G89.
Chronic Mental conditions		
Anxiety	ICD-9-CM Code: 293, 300, 308, 309, 313.	ICD-10-CM Code: F40, F41.
Bipolar disorder	Clinical Classification Code: 128.	ICD-10-CM Code: F31.
Major Depression	-	ICD-10-CM Code: F32.
Mood disorders	-	-
Pervasive developmental disorders	ICD-9-CM Code: 296, 311.	ICD-10-CM Code: F84.
Stress	Clinical Classification Code: 657.	ICD-10-CM Code: F43.
